# Intensive Lipid-Lowering Therapy Ameliorates Asymptomatic Intracranial Atherosclerosis

**DOI:** 10.14336/AD.2018.0526

**Published:** 2019-04-01

**Authors:** Huijuan Miao, Yujiao Yang, Han Wang, Linyu Huo, Mengnan Wang, Yinghua Zhou, Yang Hua, Ming Ren, Changhong Ren, Xunming Ji, Qi Yang, Xiuhai Guo

**Affiliations:** ^1^Department of Neurology, Xuanwu Hospital, Capital Medical University, Beijing, China; ^2^Department of Neurology, Sanbo Brain Hospital, Beijing, China; ^3^Department of Neurology, Huimin Hospital, Beijing, China; ^4^Department of Neurology, Haidian Hospital, Beijing, China; ^5^Department of Ultrasonography, Xuanwu Hospital, Capital Medical University, Beijing, China; ^6^Laboratory of Hypoxia, Xuanwu Hospital, Capital Medical University, Beijing, China; ^7^Department of Neurosurgery, Xuanwu Hospital, Capital Medical University, Beijing, China; ^8^Department of Radiology, Xuanwu Hospital, Capital Medical University, Beijing, China

**Keywords:** intracranial atherosclerosis, asymptomatic, transcranial color-coded sonography, lipid-lowering therapy

## Abstract

Statins have proven to exert protective effects in patients with symptomatic intracranial atherosclerotic stenosis (SICAS). It is unclear whether intensive lipid-lowering therapy (ILLT) can ameliorate atherosclerosis in asymptomatic ICAS (AICAS). A single-center, prospective cohort study was performed in 71 AICAS patients with lipid-lowering therapy. Vascular stenoses were evaluated with transcranial color-coded sonography (TCCS) before and after statin treatment. With target therapeutic level of low-density lipoprotein cholesterol (LDL-C) ≤ 1.8 mmol/L or ≥ 50% reduction from baseline after the two years of follow-up, patients were divided into intensive statin treatment (IST) group and standard statin treatment (SST) group. A total of 104 stenotic intracranial arteries were detected in 51 patients belonging to the IST group and 47 arteries in 20 patients of the SST group. In the first year, LDL-C levels were significantly decreased in the IST compared with SST groups (1.48 ± 0.26 *vs.* 2.20 ± 0.58, P=0.000). However, the ratio of regressed ICAS in IST was not significantly higher than that in SST (26.3% *vs.* 5.9%, P=0.052). Forty-nine branches in 25 patients of the IST group and 16 branches in 7 patients of the SST group were followed up for two years. The LDL-C level was decreased in the IST compared with SST groups (1.55 ± 0.29 *vs.* 2.36 ± 0.77, P=0.048). The ratio of regressed ICAS in the IST group was significantly higher than that in SST group (34.7% *vs.* 6.3%, P=0.017). We concluded that the degree of stenosis in AICAS can be ameliorated with intensive lipid-lowering therapy within two years; target LDL-C level can be reached by moderate-intensity statin treatment for Chinese AICAS patients.

Stroke has been the leading cause of death in China and is characterized by high recurrence rate and mortality. Intracranial atherosclerotic stenosis is a common cause of ischemic stroke worldwide, especially in Asian, Hispanic, and African-American populations [1-4]. Furthermore, symptomatic intracranial atherosclerotic stenosis (SICAS) is demonstrated to be a high-risk factor for subsequent stroke recurrence [5-8]. However, relevant studies focusing on asymptomatic ICAS (AICAS) are rare. The annual risk of stroke in AICAS is reported to be around 2.8% [5]. Available data showed that the prevalence of AICAS detected by transcranial Doppler was 6.9% in Chinese subjects above 40 years of age [9]. In fact, the prevalence of AICAS could increase to 29.6% for patients possessing four associated vascular risk factors namely, age, high blood pressure, high cholesterol, and diabetes [10]. Taken together, these studies indicate that AICAS is a serious area of concern, especially to the Chinese and Asian populations, and further investigation is warranted.

Statins have proven to exert protective effects in patients with cardiovascular and cerebrovascular atherosclerosis. The Stroke Prevention by Aggressive Reduction in Cholesterol Levels Study (SPARCL) indicated that high-dose statin use reduced stroke and transient ischemic attack (TIA) events by 23% [11-13]. Meta-analyses of statin treatment for stroke prevention concluded that statins reduce the incidence of stroke by about 20% with better functional improvement [14-16]. Atherosclerosis is a progressive disease, moreover, it has been confirmed in several studies that statins have a stabilizing or even reverse effect on aortic atherosclerosis [17]. Statin therapy was also associated with slower rates of progression in plaque burden and intima-media thickness among patients with asymptomatic carotid atherosclerosis [18, 19]. Extensive analysis revealed an association between the preventive effects of statins with low-density lipoprotein cholesterol (LDL-C) reduction [16]. However, most studies and medical management recommendations were for patients with SICAS, while information on the significance of statins and its efficacy as a lipid-lowering therapy in AICAS were sparse. We hypothesize that aggressive statin therapy can reverse the degree of stenosis for patients with AICAS. In this study, we aimed to explore the effects of intensive lipid-lowering therapy (ILLT) on ameliorating atherosclerosis in AICAS through a single-center, longitudinal study.

**Figure 1.**
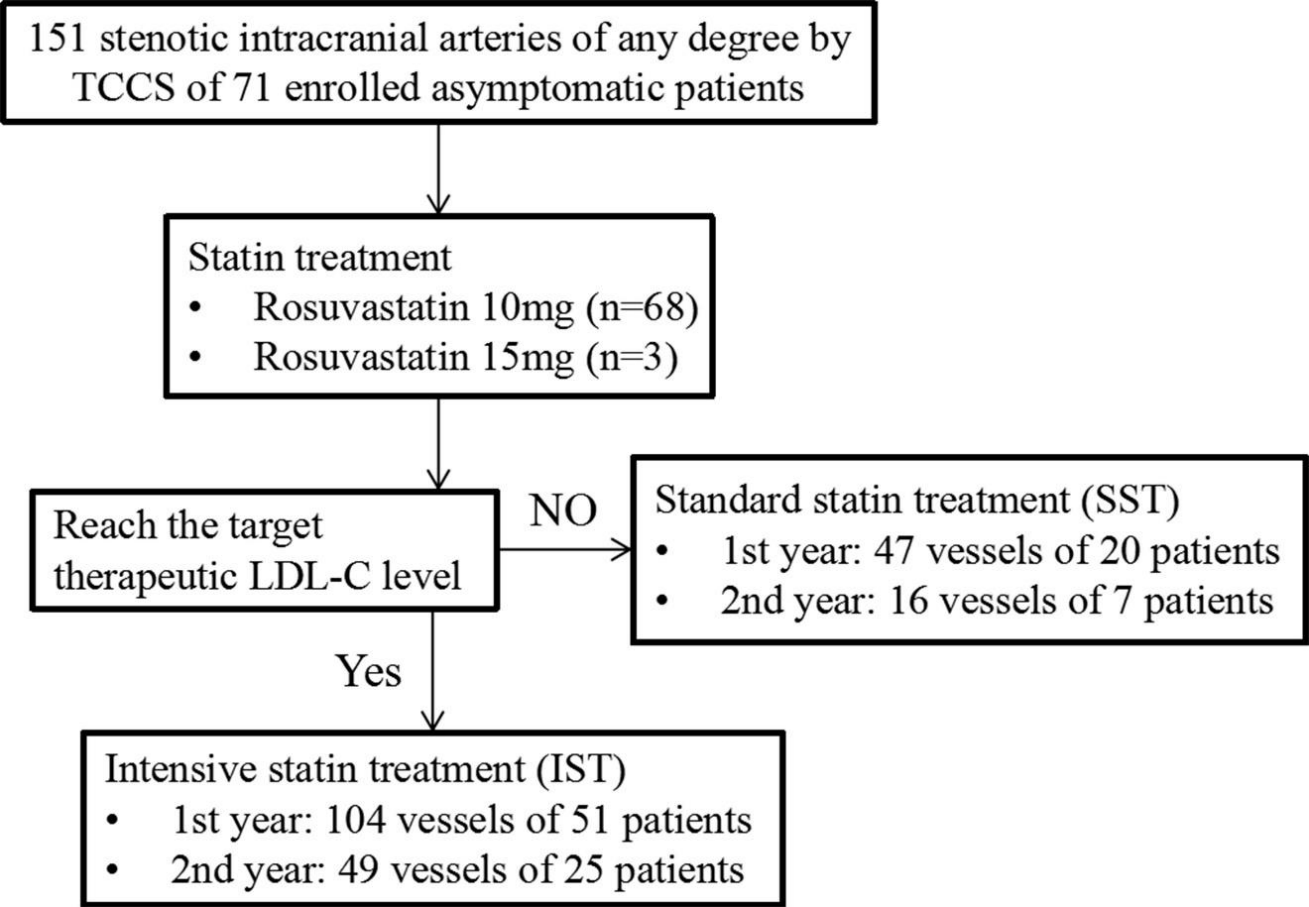
Patients and vessel selection. TCCS: transcranial color-coded sonography; LDL-C: low-density lipoprotein cholesterol.

## MATERIAL AND METHODS

### Trial Design and participants

This was a single-center and prospective cohort study approved by the institutional Medical Ethical Committee of Xuanwu Hospital. We consecutively enrolled 71 Chinese patients who came to our outpatient clinic between January 2013 and June 2016. Informed consent was obtained from all participants. The inclusion criteria were as follows: (1) 18 to 80 years, (2) any degree of intracranial artery stenosis confirmed by transcranial color-coded sonography (TCCS), (3) no history of cerebrovascular events or ischemic events occurred in vascular territory outside the affected artery, (4) at least two cerebrovascular atherosclerotic risk factors, (5) no history of lipid-lowering therapy, (6) provision of informed consent. Patients with the following conditions were excluded: (1) presence of a poor temporal acoustic window, (2) non-atherosclerotic intracranial stenosis, such as dissection, vasculitis, or Moyamoya disease, (3) presence of allergic reaction to any of the study medications, including aspirin, clopidogrel, or rosuvastatin, (4) severe liver or renal malfunction. The middle cerebral artery (MCA), terminal internal carotid artery (TICA), anterior cerebral artery (ACA), posterior cerebral artery (PCA), and basilar artery (BA) were evaluated using TCCS. Regular and long-term statin treatments were initiated once the ICAS was detected. Rosuvastatin 10-15 mg/day was given to all enrolled subjects. We followed up with the enrolled patients every 3 months by telephone asking about their physical condition, drug tolerance, blood biochemistry results, and repeated TCCS examinations for them at the end of the first and second year. The operator was unaware of patients’ clinical status. With target therapeutic level of LDL-C ≤ 1.8 mmol/L or decreased by more than 50% from baseline after the two years of follow-up, participants were divided into intensive statin treatment group (IST) and standard statin treatment group (SST). We defined the intensive statin treatment when the patient’s average LDL-C level of all his blood biochemistry tests met the final target, otherwise, it was defined as the standard statin treatment (Fig. 1).

### Diagnosis of ICAS

The examination was performed using TCCS (Asendarce, Hitachi Limited, Japan), and a 1.0-5.0 MHz Pure Wave probe was used to detect intracranial arteries (bilateral MCAs, TICAs, ACAs, PCAs, and BA). The method followed the Guidelines for Vascular Doppler Ultrasonography of China to measure and record the peak systolic velocity (PSV) and end diastolic velocity (EDV), with which the pulsatility index (PI) was calculated. The follow-up TCCS examinations were performed with the same TCCS machine and protocol. For each patient, the degree of stenosis of the intracranial arteries was graded into three categories according to the peak systolic flow velocity: mild (140-180 cm/s), moderate (180-220 cm/s), and severe (≥ 220 cm/s). We defined regression as an improvement of stenosis by one or more grades on follow-up TCCS results compared with the initial TCCS results. Conversely, we defined progression as worsening of stenosis by one or more grades. The arteries would be classified as stationary if the degree of stenosis did not change.

### Assessment of vascular risk factors

Patient clinical profile including age, sex, body mass index (BMI), diet, history of smoking and drinking, hypertension, diabetes mellitus, dyslipidemia, hyperhomocysteinemia, and coronary heart disease (CHD) were recorded. The BMI was calculated using the formula: weight (kg) divided by [height (m)]^2^. Hypertension was defined as having a history of hypertension, current use of antihypertensive medication, systolic blood pressure ≥ 140 mmHg or diastolic blood pressure ≥ 90 mmHg. Diabetes mellitus was defined as having a history of diabetes, fasting blood glucose ≥ 126 mg/dL and current use of insulin or oral hypoglycemic agents. Hyperlipidemia was defined as having a history of hyperlipidemia, current use of lipid-lowering medications, or LDL-C ≥ 130 mg/dL, triglycerides ≥ 150 mg/dL. A history of coronary artery disease includes having a history of myocardial infarction, angina, coronary angioplasty, or coronary bypass surgery.

### Assessment of outcomes

After the first TCCS examination, patients were followed for two years to detect the stenotic change under statin treatment. The primary endpoint was the regression rate of the stenotic vessels, and secondary endpoints were adverse reactions to the drug (i.e., abnormality in AST/ALT and/or serum creatine kinase level, muscle pain, related hemorrhagic events, etc.) as well as further vascular events (i.e., ischemic stroke, hemorrhagic stroke, TIA, acute coronary syndrome, death of any cause, etc.). Acute coronary syndrome was defined as acute myocardial infarction and angina with positive exercise stress test, thallium scan, or coronary angiography.

### Statistical analysis

Categorical variables were presented as frequencies and percentages, whereas continuous variables were expressed as mean ± SD. We examined the differences in discrete variables using χ^2^ and Fisher exact tests and the differences in continuous variables using a one-way ANOVA or *t*-test. Linear regression analyses were further applied to investigate the association of velocity changes of stenotic arteries showed on TCCS with the level of LDL-C. In all analyses, values of p<0.05 were deemed statistically significant. Statistical analysis was performed with the SPSS software package (version 19.0, IBM Corp, Armonk, NY, USA).

**Table 1:** Baseline characteristics of IST and SST group.

	IST (n=51)	SST (n=20)	p Value
Age (years)	59.96 ± 9.679	60.20 ± 9.715	0.926
Male (%)	24 (47.1%)	12 (60%)	0.237
BMI (kg/m^2^)	23.71 ± 6.47	24.48 ± 4.07	0.574
Smoking (%)	17 (33.3%)	9 (45%)	0.258
Drinking (%)	12 (23.5%)	4 (20%)	0.509
Hypertension (%)	36 (70.6%)	14 (70%)	0.588
Diabetes mellitus (%)	14 (27.5%)	4 (20%)	0.373
Hyperlipidemia (%)	37 (72.5%)	17 (85%)	0.216
Hyperhomocysteinemia (%)	7 (17.1%)	6 (30%)	0.203
Coronary heart disease (%)	11(21.6%)	0	0.019
Initial lipid level (mmol/L)			
LDL-C	2.51 ± 1.00	2.63 ± 0.59	0.560
HDL-C	1.38 ± 0.30	1.38 ± 0.35	0.979
NLDL-C	2.68 ± 1.01	2.99 ± 0.99	0.425
Statin level (mg/day)	10.20 ± 0.98	10.25 ± 1.12	0.840
Mean stenotic vessels	2.04 ± 1.25	2.35 ± 1.63	0.571

IST: intensive statin treatment group; SST: standard statin treatment group; BMI: body mass index; LDL-C: low-density lipoprotein cholesterol; HDL-C: high-density lipoprotein cholesterol; NLDL-C= LDL-C + VLDLC (very-low density lipoprotein cholesterol)

### RESULTS

#### Patients and Baseline Characteristics

The study cohort enrolled a total of 71 AICAS patients within the age range of 30 to 80 years old. The most common risk factor was hyperlipidemia accounting for as many as 85% of these patients, followed by a history of hypertension (70.6%) and diabetes mellitus (27.5%). The average LDL-C level at baseline was 2.51 mmol/L to 2.63 mmol/L and the mean statin level (Rosuvastatin) was 10.20 mg/day to 10.25 mg/day. The average stenotic intracranial arteries including any degree of stenosis in the two groups were 2.04 and 2.35, respectively (P=0.571), which indicated that a majority of these patients were mostly in the same risk stratification (Table 1).

The baseline characteristics of all these patients were well-balanced between the intensive and standard treatment groups. There was no statistically significant difference between the two groups with respect to age, BMI, smoking, drinking, initial LDL-C level, average stenosis, hypertension, diabetes, hyperlipidemia and hyperhomocysteinemia, except for a history of coronary heart disease (P=0.019), suggesting that patients who suffered from heart disease tended to follow doctors’ recommendations.

#### The Location and Degree of Stenotic Arteries

A total of 104 asymptomatic intracranial atherosclerotic stenotic vessels in 51 IST patients and 47 in 20 SST patients were detected with TCCS (Table 2).

In the IST group, the distributions of asymptomatic stenotic intracranial arteries were: middle cerebral artery (58.7%), terminal ICA (11.5%), anterior cerebral artery (10.6%), basilar artery (9.6%), posterior cerebral artery (9.6%). In the SST group, the proportions were: middle cerebral artery (38.3%), anterior cerebral artery (23.4%), terminal ICA (19.1%), basilar artery (10.6%), posterior cerebral artery (8.5%). There was no statistically significant difference in AICAS location between the two groups. The stenotic vessels found in the anterior circulation, including the middle cerebral artery, terminal ICA, and anterior cerebral artery, were about four times more than those found in the posterior circulation including basilar artery and posterior cerebral artery (IST 80.8% *vs.* 19.2%, SST 80.9% *vs.* 19.1%). About half of these intracranial arteries were mildly stenotic, and there was no statistical significance in different degrees of stenosis either (Table 2).

**Table 2:** Location and severity of AICAS (baseline TCCS results).

	IST (104)	SST (47)	p Value
Location of stenosis, n (%)			0.106
Anterior cerebral artery	11 (10.6%)	11 (23.4%)	
Middle cerebral artery	61 (58.7%)	18 (38.3%)	
Terminal ICA	12 (11.5%)	9 (19.1%)	
Basilar artery	10 (9.6%)	5 (10.6%)	
Posterior cerebral artery	10 (9.6%)	4 (8.5%)	
Degree of stenosis, n (%)			0.481
Mild	58 (55.8%)	23 (48.9%)	
Moderate	30 (28.8%)	13 (27.7%)	
Severe	16 (15.4%)	11 (23.4%)	

IST: intensive statin treatment group; SST: standard statin treatment group

#### Follow-up Results of LDL-C Levels at Different Years

Sixty-four patients (IST group 48, SST group 16) were followed for one year, and thirty-two patients (IST 25, SST 7) were followed for two years. The LDL-C levels at baseline were 2.51 ± 1.00 mmol/L in the IST group and 2.63 ± 0.59 mmol/L in the SST group. In the first year, both groups have a significant decrease compared with baseline (IST: 2.51 ± 1.00 mmol/L *vs.* 1.48 ± 0.26 mmol/L, p=0.000; SST: 2.63 ± 0.59 mmol/L *vs.* 2.20 ± 0.58 mmol/L, p=0.048). In addition, there was also significant difference in these two groups in the mean LDL-C levels (2.20±0.58 mmol/L *vs.* 1.48±0.26 mmol/L, P=0.000).

**Table 3:** LDL-C levels at different follow-up years.

	IST	SST	p Value
p Value	0.000	0.154	
Initial	2.51 ± 1.00	2.63 ± 0.59	0.560
First year	1.48 ± 0.26	2.20 ± 0.58	0.000
Second year	1.55 ± 0.29	2.36 ± 0.77	0.048

In the second year, LDL-C levels were significantly decreased from baseline in the IST group (2.51 ± 1.00 mmol/L *vs.* 1.55 ± 0.29 mmol/L, p=0.000), but not in the SST group (2.63 ± 0.59 mmol/L *vs.* 2.36 ± 0.77 mmol/L, p=0.379). Furthermore, there was a significant difference in the mean LDL-C levels for both groups (IST: 2.36 ± 0.77 mmol/L *vs.* SST: 1.55 ± 0.29 mmol/L, p=0.048). Follow-up results for two years illustrated the effectiveness of intensive lipid-lowering therapy for reducing the LDL-C levels (up to 0.81 mmol/L) (Table 3).

### Follow-up Results of Vascular Stenosis at Different Years

We mainly explored the regression of stenosis between the IST and SST groups at the first and second year of follow-up (Table 4).

We followed 48 patients with 95 branches of AICAS in the IST group and 16 patients with 34 branches of AICAS in the SST group for one year. In the IST group, 25 AICAS branches regressed, 62 were stable and 8 progressed. In the SST group, 2 AICAS branches regressed, 31 were stable and 1 progressed. During the first year of the follow-up period, the regression rate of stenotic vessels between these two groups were not significantly different (26.3% *vs.* 5.9%, p=0.052).

**Figure 2.**
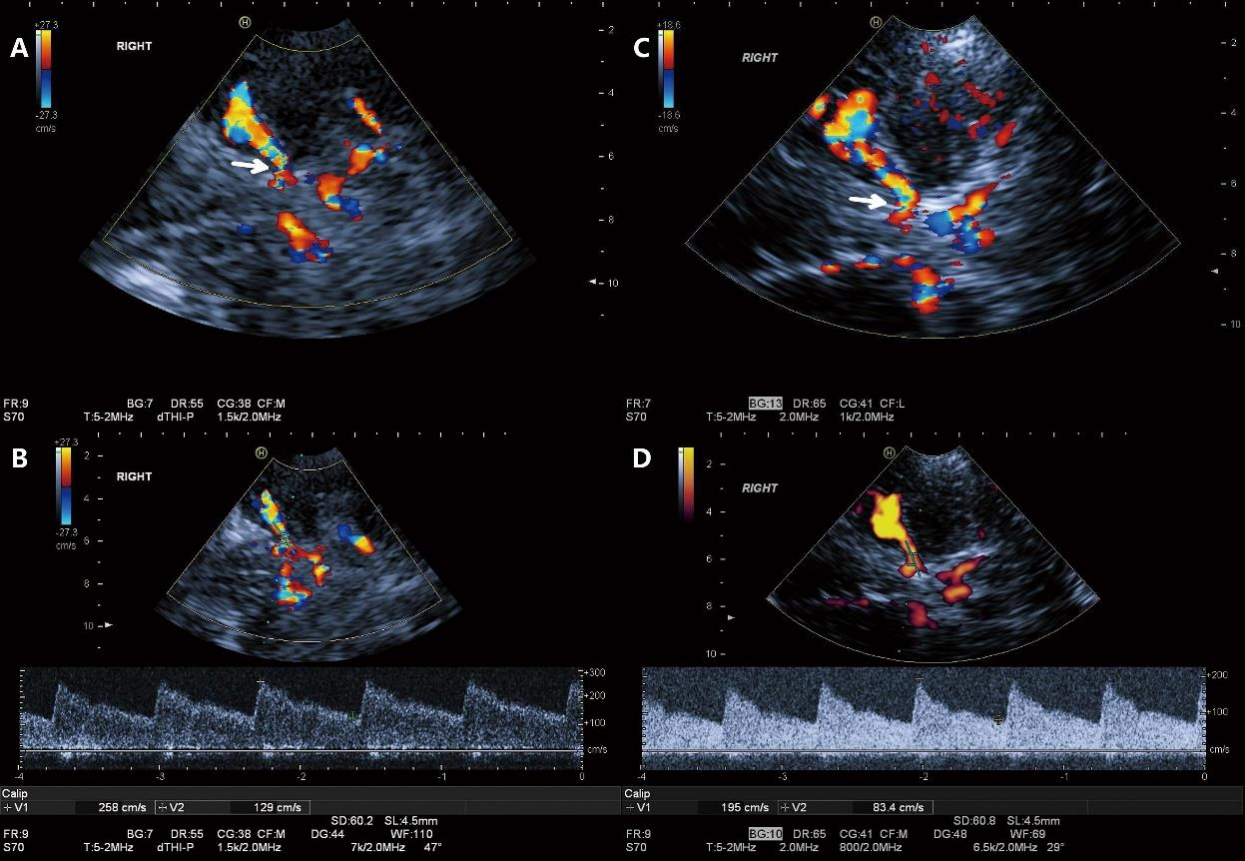
A 33-year-old man with asymptomatic intracranial atherosclerosis (AICAS) in the intensive statin treatment group (IST). A and B) TCCS at one-year follow-up showed a peak systolic velocity (PSV) of 258 cm/s for the right proximal middle cerebral artery (MCA), which indicated severe stenosis (arrow). C and D) after two years of lipid-lowering treatment of Rosuvastatin (10 mg/day), follow-up TCCS showed an obvious reduction of PSV to 195 cm/s, indicating moderate stenosis (arrow).

**Figure 3.**
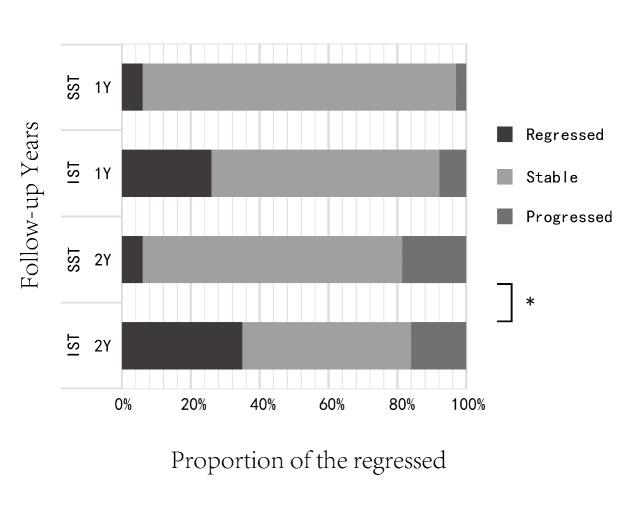
Follow-up results of stenotic vessels in the IST and SST groups in the first and second year. The regression rate in the IST group was significantly higher than that in the SST group (*p=0.017).

We followed 24 patients with 49 branches of AICAS in the IST group and 7 patients with 16 branches of AICAS in the SST group for two years. In the IST group, 17 branches regressed (Fig. 2), 24 were stable and 8 progressed. In the SST group, 1 regressed, 12 were stable and 3 progressed. After a follow-up of two years, the regression rate of ICAS in IST was significantly higher than that in SST (34.7% *vs.* 6.3%, P=0.017) (Fig. 3). The severity of the stenosis did not impact vessel development for both groups at the 1 and 2 years follow-up timepoint (Table 5). Meanwhile, there was no significant difference in the relationship between cerebral blood flow velocity change and serum LDL-C level (for the first year: p=0.181; for the second year: p=0.389).

**Table 4:** Changes in stenoses between IST and SST in the first and second year of follow-up.

First year					Second year			
	Regressed	Stable	Progressed	p Value	Regressed	Stable	Progressed	p Value
				0.106				0.091
IST (n)	25	62	8		17	24	8	
SST (n)	2	31	1		1	12	3	

IST: intensive statin treatment group; SST: standard statin treatment group

### The Safety of the Statins for the treatment of AICAS

Sixty-eight of 71 patients were treated with Rosuvastatin (10 mg/day) while the remaining were given Rosuvastatin (15 mg/day). The mean statin levels in the two groups were 10.20 ± 0.98 mg/day and 10.25 ± 1.12 mg/day. We defined severe adverse reaction to Rosuvastatin when AST and ALT levels were three times higher than normal, serum creatine kinase level was ten times higher than normal, experience of muscle pain and related hemorrhage events. During the follow-up period, a total of 3 patients were observed to have severe ALT/AST elevation (1/71) and statin-associated muscle pain (2/71). The incidence of statin-associated adverse reaction was 4.2%. There were no cerebrovascular events in any enrolled patients.

### DISCUSSION

Intracranial arterial stenosis is a progression of luminal stenosis that mainly presents with a peak systolic velocity detected with TCCS. In this study, regular re-examinations with TCCS showed that intensive lipid-lowering therapy can stabilize or even ameliorate asymptomatic intracranial atherosclerosis (P=0.017) within two years. It indicated that persistent intensive statin therapy had a significant efficacy for the regression of intracranial stenotic vessels. These findings in the development of the degree of asymptomatic stenosis were in accordance with relevant reports using ultrasound [19-23].

**Table 5:** The changes of different stenotic levels in the first and second year of follow-up.

		IST					SST				
		Regressed	Stable	Progressed	Total	p Value	Regressed	Stable	Progressed	Total	P Value
						0.998					0.777
1^st^ Year	Mild (n)	13	32	4	49		1	15	1	17	
	Moderate (n)	8	20	3	31		1	9	0	10	
	Severe (n)	4	10	1	15		0	7	0	7	
						0.454					0.031
2^nd^ Year	Mild (n)	7	9	5	21		0	9	1	10	
	Moderate (n)	7	8	3	18		0	2	2	4	
	Severe (n)	3	7	0	10		1	1	0	2	

IST: intensive statin treatment group; SST: standard statin treatment group

Our findings have significant clinical value. The Chinese Intracranial Atherosclerosis (CICAS) Study showed that ICAS is the most common vascular lesion in patients with cerebrovascular disease in China [24-26]. The prevalence of asymptomatic MCA stenosis has been reported to be as high as 12.6% [10]. However, aggressive medical intervention was not recommended because of its relatively low risk for stroke. A prospective cohort study found a prevalence of AICAS in stroke-free Caucasians with moderate-high vascular risk (8.6%) [27]. Another longitude cohort study demonstrated that even mild to moderate AICAS is an independent risk factor for future ischemic stroke in a healthy population [28]. The significance of AICAS as well as treatment strategies remains a matter of debate. This study showed that even a moderate dosage of statin could effectively decrease stenotic levels in two years.

In addition to lowering LDL-C, statins have been demonstrated to decrease the risk of cardiovascular and cerebrovascular events by reducing systemic inflammation and improving endothelial function [28-30]. However, reports on the effects of intensive statin use in patients with AICAS are scarce. In this study, the level of LDL-C between the two groups is significantly different after 12 and 24 months of intensive therapy. Our results indicated that LDL-C ≤ 1.8 mmol/L or decrease in more than 50% of LDL-C from baseline can be reached by administering a moderate dose of statin for Chinese patients. Chung et al. [31] demonstrated that premorbid statin usage is independently associated with reduced plaque enhancement, which is in accordance with its effects in coronary artery disease [20]. ILLT could slow the progression of AICAS or even induce regression. A long-term longitudinal study with medical intervention is still needed to determine the optimal strategy for preventing stroke. More modality studies such as high-resolution magnetic resonance imaging that accurately measures the regression of stenotic arteries will be used in our future study. A longitudinal investigation would be highly valuable to confirm the effect of ILLT for AICAS.

### Limitations

We explored the relationship between the velocity difference and LDL-C level at the first and second year of follow-up in the IST group. However, no linear relationship was found. This may attribute to various factors affecting velocity measurement using TCCS, which includes hematocrit, temperature, levels of arterial oxygen and operator’s skills.

### Conclusions

We conclude that: (i) the degree of atherosclerosis in AICAS is further reduced with intensive lipid-lowering therapy; (ii) LDL-C ≤ 1.8 mmol/L or more than 50% decrease in LDL-C can be reached by administering a moderate dose of statin for Chinese patients with AICAS.
